# Relative Copy Number Variations of *CYP2C19* in South Indian Population

**DOI:** 10.1155/2012/643856

**Published:** 2012-06-25

**Authors:** Anichavezhi Devendran, Chakradhara Rao Satyanarayana Uppugunduri, Rajan Sundaram, Deepak Gopal Shewade, Krishnamoorthy Rajagopal, Adithan Chandrasekaran

**Affiliations:** ^1^Pharmacogenomics Laboratory, Department of Pharmacology, Jawaharlal Institute of Postgraduate Medical Education and Research, Pondicherry 605006, India; ^2^Onco-Hematology Unit, Department of Pediatrics, University Hospital of Geneva, Geneva, Switzerland; ^3^U 763, INSERM, Hopital Robert Debre, Paris, France

## Abstract

CYP2C19 is a polymorphic enzyme involved in the metabolism of clinically important drugs. Genotype-phenotype association studies of *CYP2C19* have reported wide ranges in the metabolic ratios of its substrates. These discrepancies could be attributed to the variations in the promoter region and this aspect has been reported recently. The observations in the recent reports on the influence of promoter region variants on the metabolism of CYP2C19 substrates might also have been influenced by the copy number variations of *CYP2C19*. In this paper, we describe copy number variations of *CYP2C19* using real-time polymerase chain reaction by comparative Ct method. No copy number variations were observed in the south Indian population indicating the observed discrepancies in genotype-phenotype association studies might be due to the regulatory region polymorphisms as reported earlier.

## 1. Introduction

The CYP2C19 is a clinically important drug metabolizing enzyme encoded by polymorphic* CYP2C19* gene on chromosome 10, playing a major role in metabolizing about 5% of the clinically used drugs [1–5]. Owing to genetic polymorphisms, considerable interindividual variability exists in the metabolic activity of this enzyme [6]. About 43 variant alleles of *CYP2C19* have been reported till date (http://www.imm.ki.se/CYPalleles, access date: 28th March 2012). Most of the genotype-phenotype association studies of *CYP2C19 *in different ethnic groups were focused on exonic region variants, but the promoter region variants and copy number variations were not explored to a greater extent [7–11]. Based on the findings of genotype-phenotype association studies, subjects were categorized as poor metabolizers (PMs), having either of the most commonly seen defective alleles of *CYP2C19*, namely, c.636G>A (*3 allele; rs4986893) or c.681G>A (*2 allele; rs4244285); extensive metabolizers (EMs) carrying no variant alleles; ultra-rapid metabolizers (UMs) carrying −806C>T and −3402C>T variations (*17 allele) in the promoter region of *CYP2C19* [12]. Thus, the activity of CYP2C19 varies with the presence or absence of certain variations in its gene which also varies in its distribution among different ethnic groups [8, 13–18].

CYP2C19 activity differs among extensive metabolizers (EM), demonstrated by a wide range in the metabolic ratios (MR) of probe drugs, and some discrepancies were commonly noted in these reports [11–20]. These differences could be attributed to rare defective alleles or to polymorphisms in the regulatory region of *CYP2C19* gene and copy number variants of the gene [20, 21]. Few studies have reported the promoter region variations of *CYP2C19* in different populations [13, 14, 22–24]. Functional characterization of the promoter region polymorphisms has been performed in a recent study from our laboratory describing the influence of promoter region polymorphisms on proguanil oxidation to an active compound mainly by CYP2C19 [23, 24]. In this paper we concluded that the discrepancies observed in genotype-phenotype association studies of *CYP2C19* were due to promoter region polymorphisms influencing the expression of CYP2C19. However, the existence of copy number variations and the variability in the basal levels of expression of CYP2C19 between individuals was not considered, which might have affected the observations. Thus an alternative explanation for these discrepancies could be the copy number variations of this gene. Therefore, here in this paper we describe the results of an explorative study analyzing copy number variations of *CYP2C19 *in the study subjects of the previous report.

## 2. Methods

The relative copy number was estimated using a real-time PCR (comparative  ΔΔCt  method of relative quantification) approach based on existing methodologies with gene-specific primers using *IL-2* as reference gene using genomic DNA obtained from the peripheral white blood cells [25, 26]. Cycle threshold (Ct) is defined as the number of cycles required for the fluorescent signal to cross the threshold, that is, exceeding the background level. The efficiency and specificity of primer pairs for *CYP2C19* (F: 5′-GCC ATT TCC CAC TGG CTG AAA G-3′; R: 5′-ACG AAA CTA GGA GGG AG TCC-3′) and *IL-2* (F: 5′-TGC ACA TAA TCT CAT CTT TCT AAC ACT CTT-3′; R: 5′-TTG AAA GCG CAA TAG ATG GAC AT-3′) from genomic sequence were evaluated using serial 10-fold dilutions of human genomic DNA (*n* = 50) ranging from 0.2 to 200 ng. The demographic details of the subjects were discussed elsewhere [23]. The primers used for *CYP2C19* amplification are specific to prevent the amplification of other homologous genes such as *CYP2C9* located on the same chromosome. Total reaction volume consists of 25 *μ*L and contained the following: 12.5 *μ*L of 2X SYBR Green Master Mix (Sigma-Aldrich, St. Louis, USA), human gDNA (0.2 to 200 ng with equal volume, that is, the total amount in 2 *μ*L) and the appropriate primer pair (400 nM). The PCR thermoprofile consisted of initial activation of the polymerase at 95°C for 15 min followed by 40 PCR cycles of 95°C for 15 s, and 60°C for 1 min (40 cycles) followed by a melting curve to verify the presence of a single amplification product. Assays were performed in duplicate on an Applied Biosystems 7300 Fast Real-Time PCR system using genomic DNA of 2 *μ*L (approximately 20 ng). The PCR was performed in a 96-well clear optical reaction plate MicroAmp (Applied Biosystems). *IL-2 *gene was coamplified with *CYP2C19* gene and served as internal standard (reference gene). *CYP2C19 *copy number was calculated relative to the human gDNA sample with no genetic variations in exonic and promoter regions, showing a good genotype-phenotype correlation (standard). The following equation was used: relative copy number = 2∗2^−ΔΔCt^, where  −ΔΔCt = (Ct  of reference gene −  Ct  of target gene) of standard − (Ct  of reference gene −  Ct  of target gene) of sample. Mean Ct values were taken for each sample derived from duplicate reactions. Efficiency of the assay was calculated using the formula *E* = 10^(−1/slope)^ − 1 × 100. The calculated relative copy numbers were confirmed by recalculating the haploid copy number from the observed  *C*_*t*_  values, and then multiplied by 2 to give the diploid copy number. The alternate formula used for calculation of haploid copy number was 2^−ΔΔCt^ = (1+*E*)^−ΔCt_test  gene_^/(1=*E*)^−ΔCt_ reference  gene_^, where *E* = Efficiency of the PCR reaction;  ΔCt_ test  gene_  = Difference in the threshold cycle value between the test sample and calibrator sample for the gene (test gene) under investigation and  ΔCt_ reference  gene_  = Difference in the threshold cycle value between the test sample and calibrator sample for reference gene.

GraphPad Instat version 3.06 (San Diego, USA) was used for statistical analysis. Data were expressed as mean ± SD. The copy number range from 1.7–2.1 was considered as the normal diploid copy number.

## 3. Results and Discussion

The efficiency of the assay for *IL-2* and *CYP2C19 *was between 87–100% and 94–100%, respectively. The efficiencies were similar and the assay stability was good. The slope of the standard curve for *IL-2* was in the range of −2.7 to −4.3 with *R* ^2^ (mean) of 0.99. The slope of the standard curve for *CYP2C19 *was in the range of −3.2  to −4.5 with *R* ^2^ (mean) of 0.99. The product sizes for *CYP2C19* and *IL-2* were 193 bp and 93 bp, respectively. Dissociation curves have shown the specific melting temperatures for both *IL-2* and *CYP2C19* products and/or any primer dimer formed during amplification. The mean Ct values observed for the *CYP2C19* and *IL-2* standards are given in Table 1. The mean Ct values of *CYP2C19* and *IL-2* from healthy volunteers are given in Table 2. The amplification plots, dissociation curves, and standard curves of *CYP2C19 *and *IL-2* are given in the Supplementary Material available online at doi: 10.1155/2012/643856. No copy number variations of *CYP2C19* were detected in south Indian population. All the samples studied were found to have two copies of the gene. The *CYP2C19* copy numbers were in the range of 1.7–2.17 with slight deviation from the set value 1.7–2.1 (Table 2). The values were obtained after taking mean values of ΔΔCt from 2 reactions of the same sample and calculating copy number of each sample using formula  2∗2^−ΔΔCt^. The results of this study are in accordance with the observations made by using comparative genomic hybridization technique with bacterial artificial chromosome of an individual (personal communication from Manipal Life Sciences Center, Manipal, India).

Absence of the copy number variations of *CYP2C19* in south Indian population indicates that the genetic variations such as single-nucleotide polymorphisms may predict the changes in the activity of the enzyme. This observation also indicates that the discrepancies in genotype-phenotype association studies and wide ranges in the metabolic ratios of CYP2C19 substrates in EMs are due to other variations in the regulatory region. The involvement of other CYP isoforms in the metabolism of CYP2C19 substrates may also contribute to the discrepancies observed in genotype-phenotype association studies. Further studies are needed on a larger scale to investigate the copy number variations of *CYP2C19* in different disease states in comparison to the healthy volunteers.

## 4. Conclusion

We here by conclude that no copy number variations of *CYP2C19* were found in south Indian population suggesting that the previously observed discrepancies of phenotype-genotype studies could be due to the variations in the regulatory region as reported earlier.

## Supplementary Material

The amplification plots for *CYP2C19, IL-2* gene amplification are depicted in the supplementary figures 1 and 2 (where x-axis represents the cycle number and y-axis represents ΔRn reflecting the magnitude of the signal generated by PCR conditions used for the experiment). The dissociation curves specific for *CYP2C19* and *IL-2* amplification are given in the supplementary figures 3 and 4 (where x-axis represents temperature in °C and y-axis represents the derivative of fluorescence with respect to temperature). The standard curves for *CYP2C19* and *IL-2* gene quantification are given in the figures 5 and 6 (where x-axis represents the log transformed concentrations of the standards used and y-axis represents the cycle threshold (Ct) reflecting the cycle number at which the fluorescence generated within a reaction crossing the threshold level.

## Figures and Tables

**Table 1:** Mean Ct values for standard *CYP2C19* and *IL-2* gene amplification.

Amount of genomic DNA in standards (ng)	*CYP2C19* Ct value (mean ± SD; *n* = 2)	*IL-2* Ct value (mean ± SD; *n* = 2)
200	20.77 ± 0.084	22.55 ± 0.19
20	23.59 ± 0.076	NA
2	26.43 ± 0.092	28.81 ± 0.092
0.2	28.90 ± 0.074	32.30 ± 0.096

NA: not included in the analysis. The standard curve was obtained using three points.

**Table 2:** Mean Ct values of *CYP2C19* and *IL-2* genes amplified from genomic DNA of 50 healthy volunteers.

Ct value for *CYP2C19* (mean ± SD; *n* = 50)	Ct value for *IL-2* (mean ± SD; *n* = 50)	Number of copies calculated from 2^−ΔCt^ (range)
22.23 ± 0.995	25.15 ± 0.965	1.7–2.171
