# Supplementary material for: Relative Copy Number Variations of CYP2C19 in South Indian Population
Source: Mol Biol Int. 2012 Jun 25;2012:643856. doi: 10.1155/2012/643856 (PMC3389726; doi:10.1155/2012/643856)
Supplement: Supplementary file 1 — The amplification plots for CYP2C19, IL-2 gene amplification are depicted in the supplementary figures 1 and 2 (where x-axis represents the cycle number and y-axis represents ΔRn reflecting the magnitude of the signal generated by PCR conditions used for the experiment). The dissociation curves specific for CYP2C19 and IL-2 amplification are given in the supplementary figures 3 and 4 (where x-axis represents temperature in °C and y-axis represents the derivative of fluorescence with respect to temperature). The standard curves for CYP2C19 and IL-2 gene quantification are given in the figures 5 and 6 (where x-axis represents the log transformed concentrations of the standards used and y-axis represents the cycle threshold (Ct) reflecting the cycle number at which the fluorescence generated within a reaction crossing the threshold level. [file 643856.f1.doc]

**Supplementary information:**

**Title: Relative copy number variations of *CYP2C19* in South Indian Population**

**Authors**: Anichavezhi Devendran,^1^ Chakradhara Rao Satyanarayana Uppugunduri, ^1,2^ Rajan Sundaram, Shewade Deepak Gopal,^1^ Krishnamoorthy Rajagopal, ^3^ Adithan Chandrasekaran.^1^

^1^Pharmacogenomics Laboratory, Department of Pharmacology, Jawaharlal Institute of Postgraduate Medical Education and Research, Pondicherry 605006, India

^2^ Department of pediatrics, Onco-hematology unit, University Hospital of Geneva, Geneva, Switzerland

^3^ U 763, INSERM, Hopital Robert Debre, Paris, France.

**
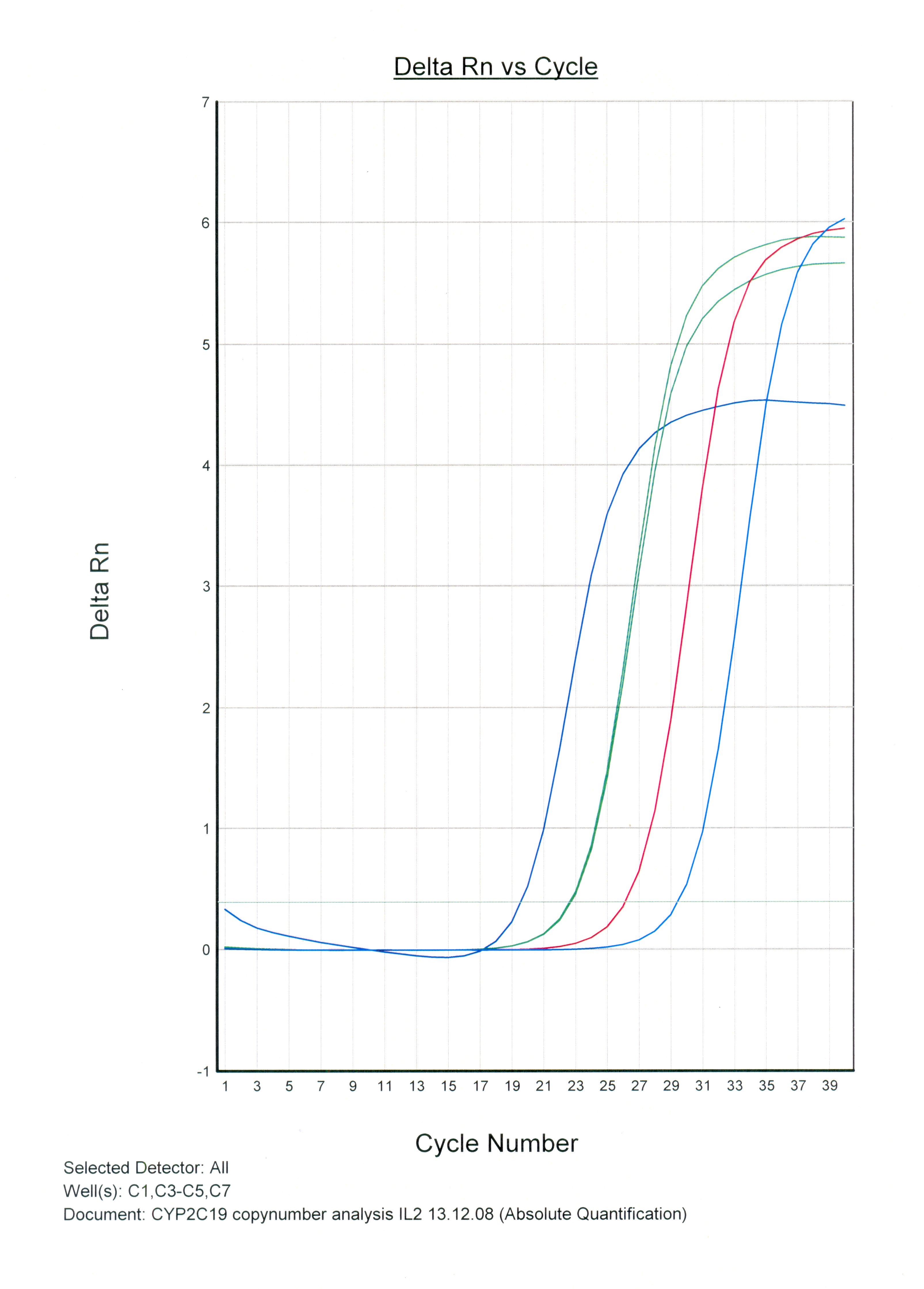
**

**Figure 1. Amplification plot for *CYP2C19* gene using template of different amount in 10 fold dilution range (from left to right the amount of template is 200, 20, 2, and 0.2 ng respectively).**

**
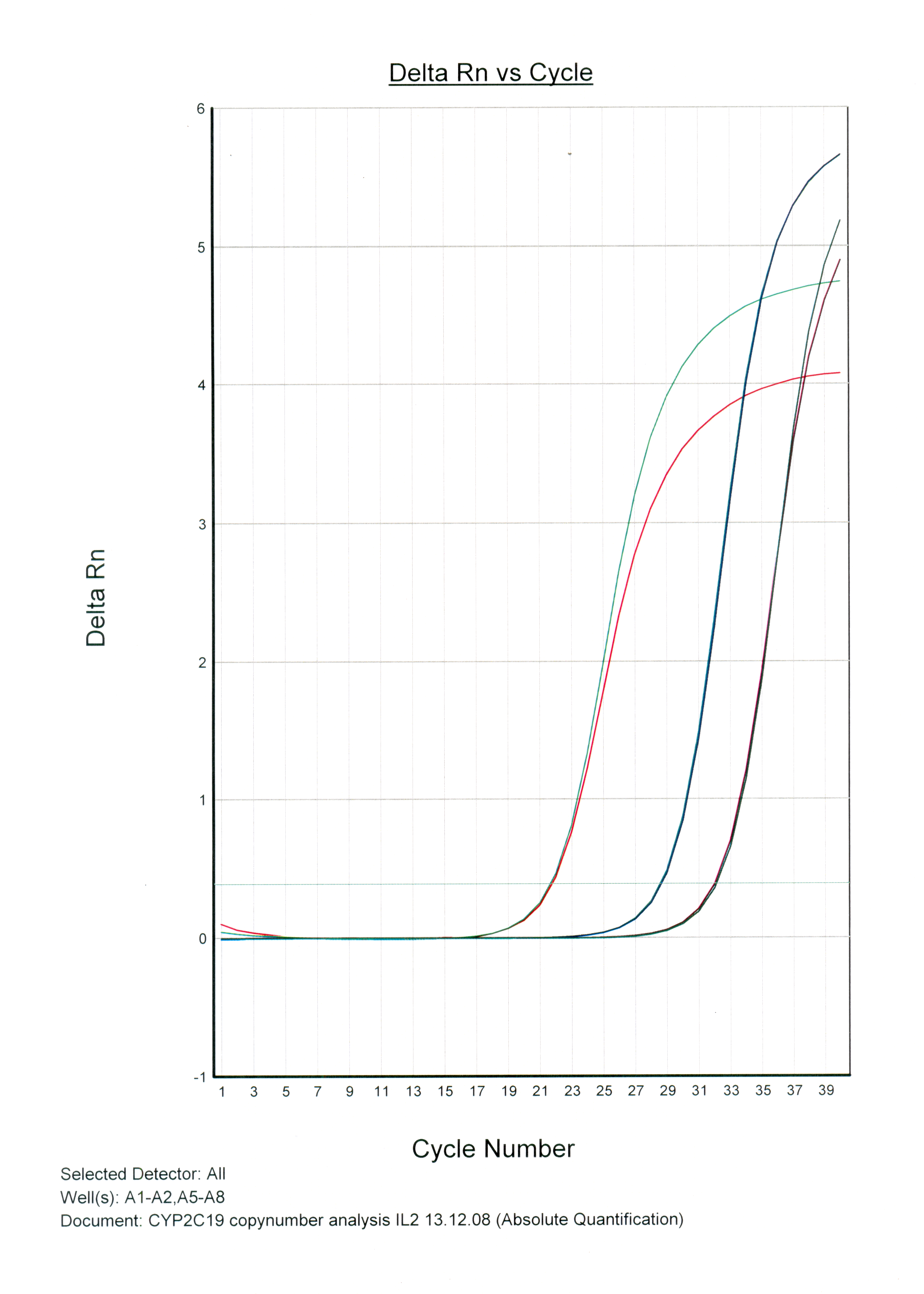
**

**Figure 2. Amplification plot for *IL-2* gene using template of different amount in 10 fold dilution range (from left to right the amount of template is 200, 2, and 0.2 ng respectively).**

**
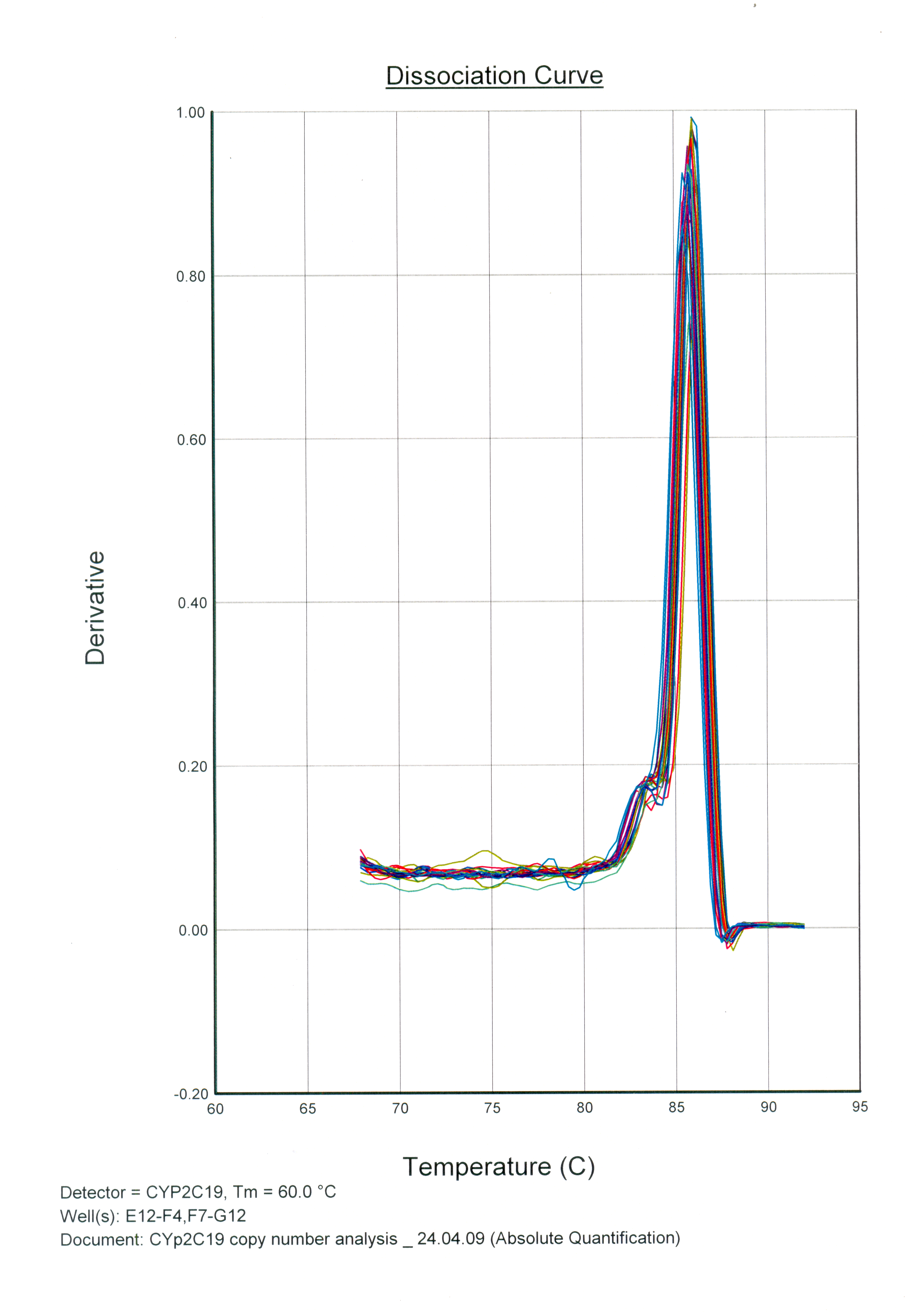
**

**Figure 3. Dissociation curve for *CYP2C19* gene amplification**

**
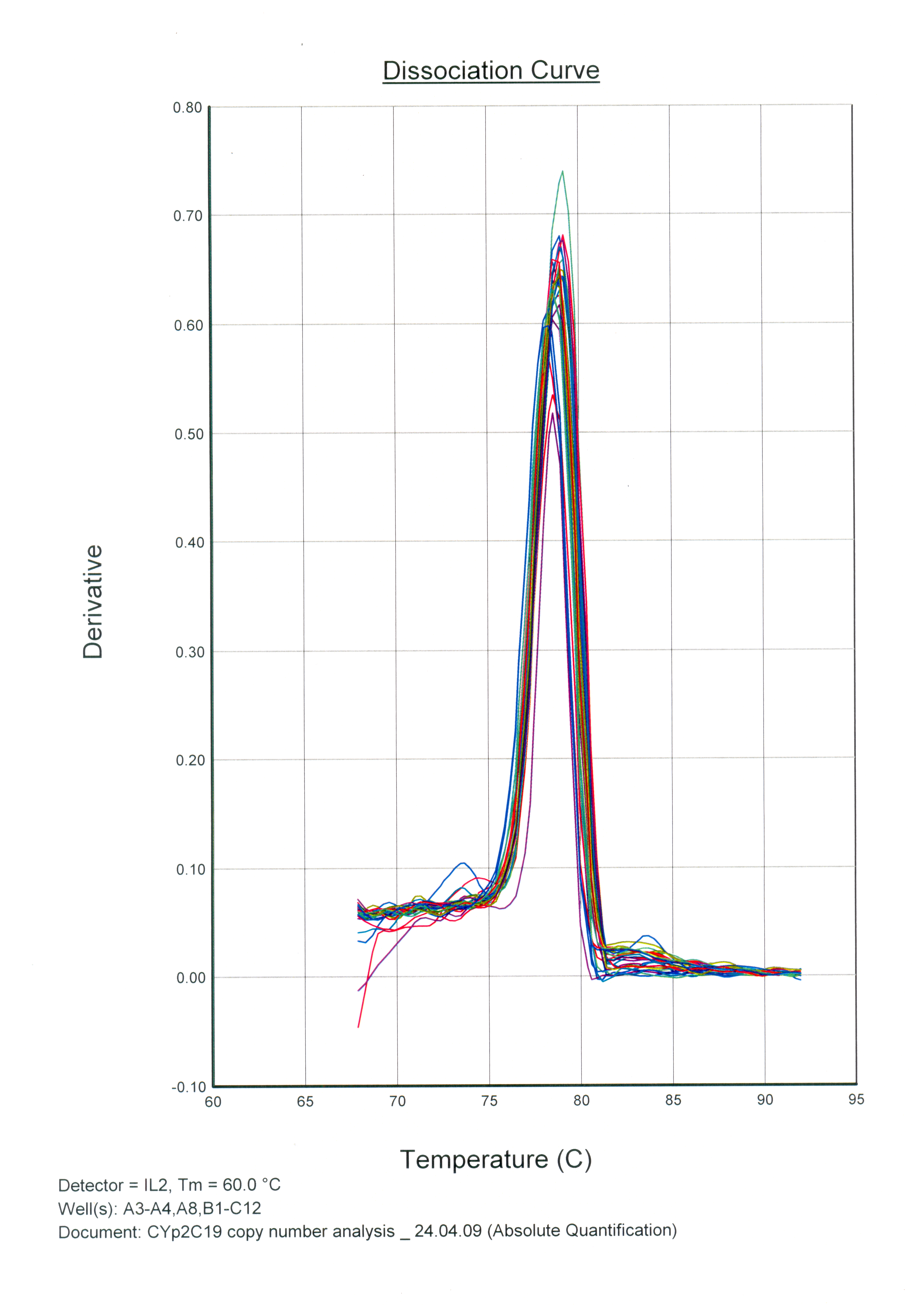
**

**Figure 4. Dissociation curve for *IL-2* gene amplification**

**
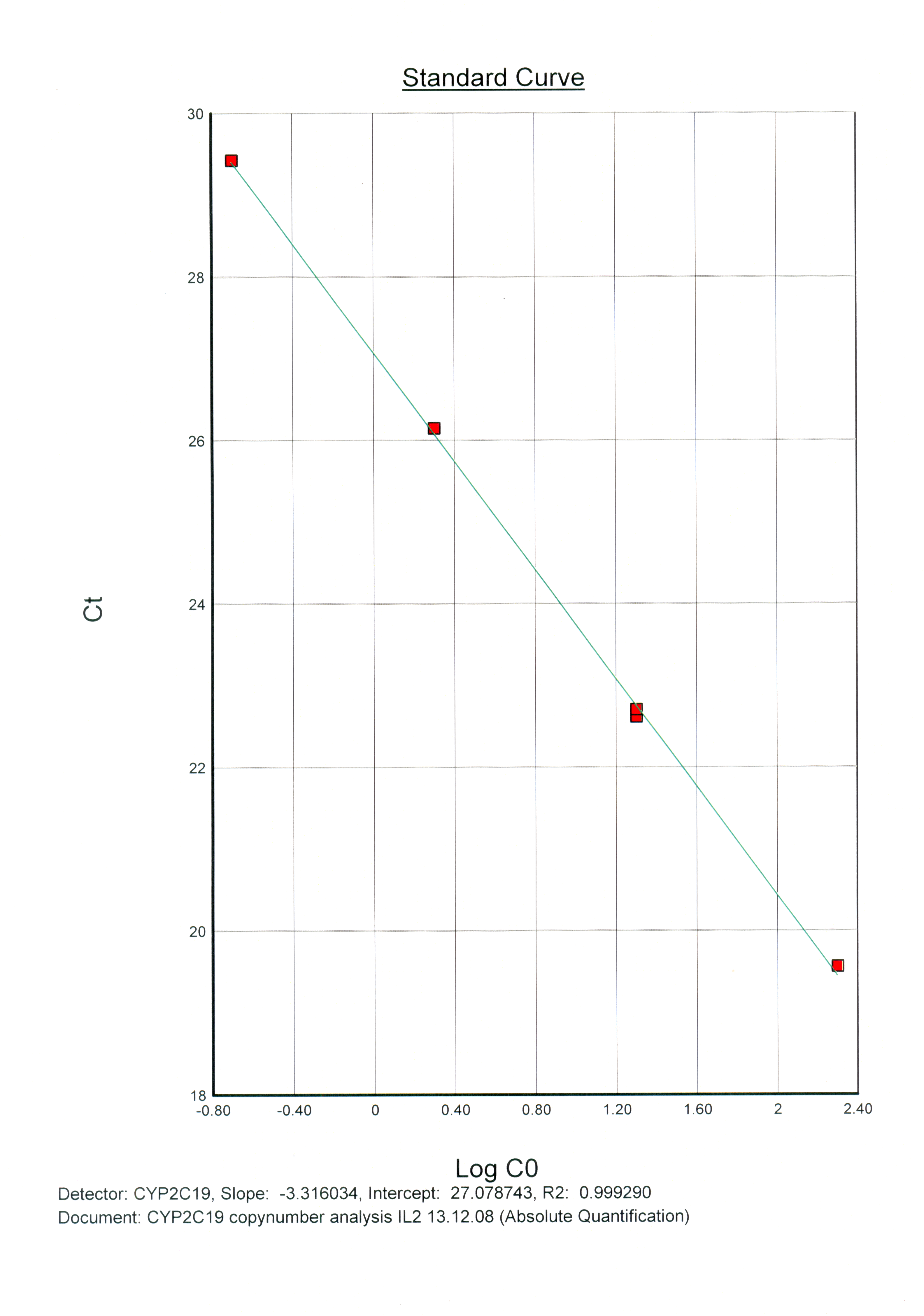
**

**Figure 5. Standard curve for *CYP2C19* gene. X-axis represents the quantity of template (Logarithmic scale) used and Y-axis represents the Ct values (Threshold for amplification).**

**
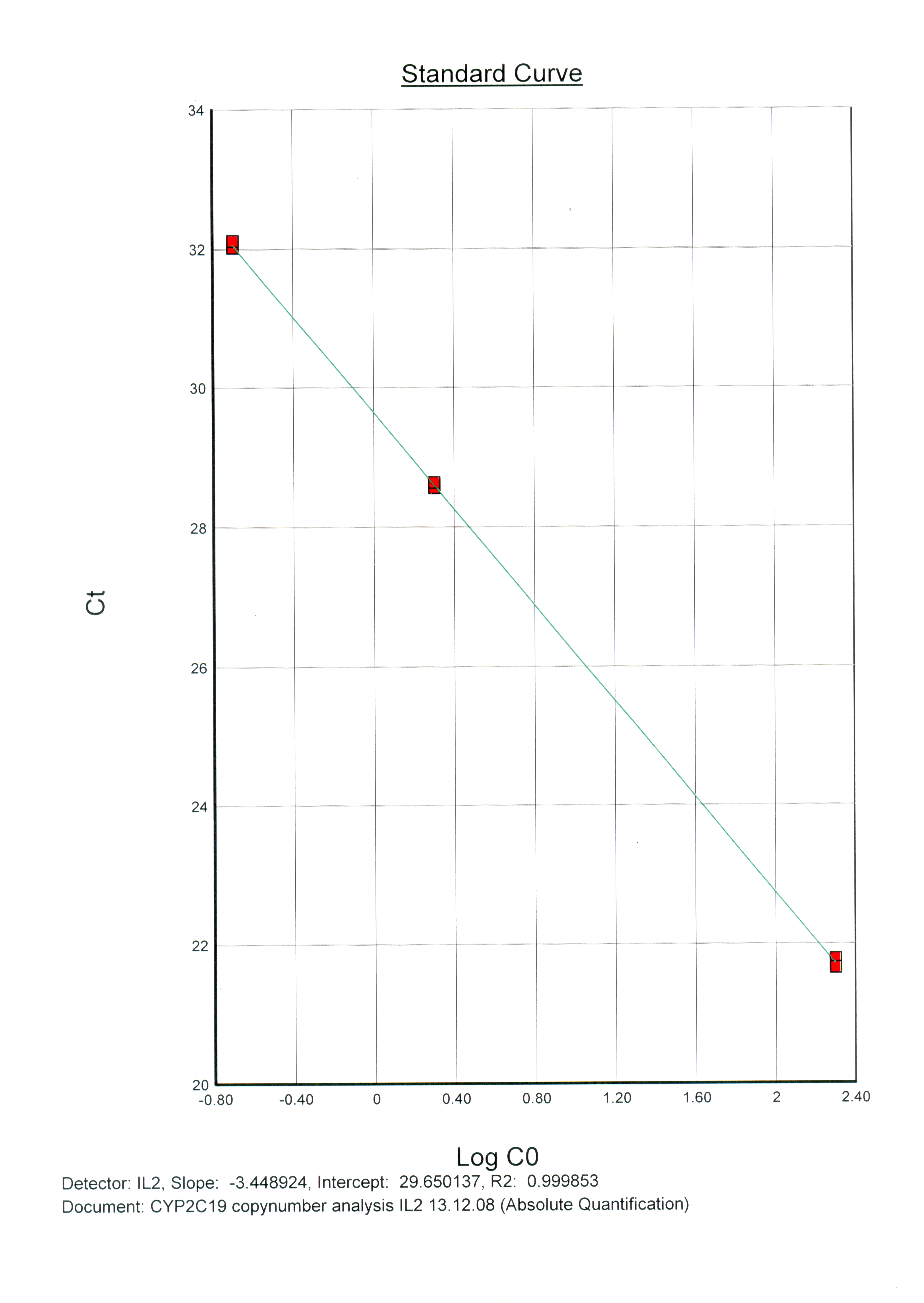
**

**Figure 6. Standard curve for *IL-2* gene. X-axis represents the quantity of template (Logarithmic scale) used and Y-axis represents the Ct values (Threshold for amplification).**

**Interpretation:** The efficiencies were similar for both internal control and the target gene amplification using the defined set of primers. Hence, comparative Ct method could be implemented for analysis of copy number of the target gene.
